# ‘It’s made me feel less isolated because there are other people who are experiencing the same or very similar to you’: Men’s experiences of using mental health support groups

**DOI:** 10.1111/hsc.13788

**Published:** 2022-03-16

**Authors:** Alex Vickery

**Affiliations:** ^1^ School for Policy Studies University of Bristol Bristol United Kingdom; ^2^ School of Social Sciences Cardiff University Cardiff United Kingdom

**Keywords:** distress, help‐seeking, masculinity, men's mental health, mental health, peer support, support group

## Abstract

This article explores men's experiences of using peer support groups for coping with mental distress. Support groups are organised groups in which people come together to mutually support each other with a shared health concern. There has been increasing research on men's mental health help seeking, but men's use of support groups for mental health difficulties, and the ways support groups could benefit men, is not well understood. Drawing upon 19 interviews from a South Wales, UK qualitative study which explored men's mental health help seeking, coping and management, this article explores the perceived benefits of support groups for men experiencing emotional difficulties. Findings highlight how men who attended groups valued the sense of shared understanding of experiences and the mutual respect that group settings presented them with. Support groups provided a safe space with opportunities to reconstruct traditional masculine norms through reciprocating unique and tailored mental health support to others and developing a certain role within that group. This gave men a sense of purpose which further facilitated mental health management. Findings also indicated the social benefits that support groups can have to men who may have limited social networks or be experiencing isolation. This article adds to the growing literature that focuses on men's mental health experiences and illustrates the benefits of support groups for men in distress. The author suggests that primary services need to be aware of how support groups can positively support men and promote them as an opportunity for connection and unique support.

**What is known about this topic:**

- Men are traditionally less likely to seek help for mental health difficulties than women.
- Peer support can have an impact on men's quality of life, including social benefits such as reducing isolation.
- Research on men's help seeking and support strategies focuses on initial help seeking from a General Practitioner (GP) or mental health services, having often overlooked the potential of support groups for men.

**What this paper adds:**

- Evidence that men experiencing mental health difficulties can, and do, benefit from using support groups for managing their mental health and wellbeing.
- Qualitative evidence about the benefits and positive influence support groups can have for men experiencing distress.

## 1 INTRODUCTION

There is a dominant narrative that men do not seek help for problems when experiencing distress and emotional troubles. Men continue to be three times more likely to take their own lives than women (ONS, 2020). Male mental health and suicide is complex, but much work has consistently pointed to men's reluctance to seek help (Addis & Mahalik, 2003; Oliffe et al., 2019) and how they may express and deal with mental health issues differently to women (Brownhill et al., 2005).

When men do seek out professional support, they might be limited to the general practitioner (GP) options of prescribed medication or long counselling or cognitive behaviour therapy (CBT) waiting lists. Furthermore, when men attend therapy with psychologists/psychotherapists, the therapy they receive might not be perceived as very male friendly or gender sensitive (Seidler et al., 2020). As an alternative, people presenting to the GP with distress may be offered or signposted to group support, as opposed to individual talking‐based therapies. Alternatively, they might pursue such interventions themselves. The National Health Service (NHS) and many Third Sector charity organisations have developed support group services for people experiencing emotional difficulties as a way to increase access to mental health support and meeting a preference for own self‐management. Peer support, which is often delivered through the use of groups, ‘is a system of giving and receiving help founded on key principles of respect, shared responsibility, and mutual agreement of what is helpful’ (Mead, 2003). Groups can be professionally run but are often based on peer‐to‐peer interactions. Peer support group services have been found to increase recipients’ sense of autonomy, self‐efficacy, belonging and hopefulness while decreasing symptoms of distress (Southall et al., 2019). There has been research specifically on men's use of cancer support groups (Oliffe et al., 2009, 2011) yet research on the effectiveness of group support specifically for men experiencing mental health difficulties is limited. It is unknown how this alternative means of support can be beneficial to men experiencing distress, particularly given the lower rates of help seeking and the stigma attached to talking about mental health for men.

Galdas et al. (2015) carried out a systematic review and meta‐ethnography to identify how effective, cost‐effective, accessible and acceptable self‐management support interventions such as groups are for men with long‐term health conditions (including depression). Self‐help interventions that included physical activity, education and peer support were revealed as more beneficial to men than to women and having a positive impact on men's quality of life when living with a long‐term condition. Their analysis of qualitative studies established four interconnected concepts relating to men's experiences of self‐management support, including: need for purpose, trusted environment, value of peers and becoming an expert. Cramer et al. (2014) aimed to establish if men do attend therapeutic/support groups for depression, as well as what types of groups they might attend and why. Although a small‐scale study, the results evidence that some men attend groups for support with depression and anxiety and appreciated accessing mental health support outside their immediate family and friends. Additionally, Cramer et al. (2014) found that their research participants also frequently mentioned feeling lonely, isolated or bored as motivation for attending groups.

Support groups are thought to offer social benefits in reducing social isolation and managing emotional difficulties, particularly in older men (Broughton et al., 2017). There is now a growing number of community‐based organisations that run groups specifically aimed to tackle loneliness and social isolation in older men. Research has highlighted how effective such groups can be because of the social support, relationships, social engagement and connectedness that they can provide to men who may be retired, widowed or living alone (Broughton et al., 2017; Wilson & Cordier, 2013). In Milligan and colleagues’ review of gendered interventions for older men, they found that in these men's community groups, men valued the sense of contributing rather than being clients (Milligan et al., 2016).

Simpson and Richards (2019) explored ways in which men facing disadvantage because of socioeconomic hardship and/or mental health difficulty, know, do and feel about their health and wellbeing, and how involvement in self‐help groups can support this. Participants’ accounts indicated how positive mental health was aided by support from men sharing experiences in an informal and nonclinical way. Their research highlights the significance of groups in providing emotional resources that help to normalise discomforting, emotional talk, and groups can present opportunities for supporting other men which further functioned as ongoing therapy. Likewise, Mackenzie et al. (2017) explored attendees’ discussions at men's only groups and explored whether these discussions were complicit or counter to hegemonic views of masculinity. They found the need for male focused spaces that provide opportunity for personal discussions often to do with health and mental health issues, connection through male friendly‐banter and interests and activities that were unique to men. They identified this as a counter narrative to men's previous discussions of how they, and other men, have difficulty opening up about emotional problems.

The research discussed so far aligns with the concept of ‘communities of practice’ (Lave & Wenger, 1991) that refers to how identities are learned and produced within various subgroups and locales. To identify a social context as a community of practice there must be, a mutual interaction of members, a common purpose driving this interaction and shared repertoire such as common discourses and behaviours (Wenger, 1998). A community can be broadly defined as groups or networks of people with shared understandings of norms, identity and social practices (Creighton & Oliffe, 2010). Using this framework, Simpson and Richards (2019) note how the norms that underpin self‐help support groups enabled the men in their study to express emotions that might in other contexts compromise masculine status, particularly for working class men. Thus, communities of practice in the form of support groups can assist the restructuring of masculinity in more health‐promoting ways. Similarly, McKeown et al. (2015) emphasised men's greater satisfaction with projects such as self‐help groups that were experienced as empowering compared to traditional mental health services.

### 1.1 Aim of the study: Men's experiences of using support groups for mental distress

It can be recognised that support groups can provide specific social relationships and unique social support to men in times of distress. Although not specific to mental distress, theoretical literature provides argument for how social relationships and social support improve psychological wellbeing (Thoits, 1995, 2011a, 2011b). Thoits (2011a) demonstrates how support groups produce strong social relationships, ties and support, because they can provide a safe space to be around similar people, who have had similar experiences and can offer specific advice and support. Similarly, Seebohm et al. (2013) found that self‐help groups made a strong contribution to members’ mental wellbeing by enhancing a sense of self‐esteem and control and decreasing isolation through participation. Ussher et al. (2006) identified support groups as being positioned as providing strong feelings of belonging, acceptance and a sense of community. These studies (Seebohm et al., 2013; Ussher et al., 2006) refer to support groups for health problems other than mental distress. This paper, therefore, aims to explore further men's experiences of using support groups for mental distress and the perceived effectiveness of groups for men.

## 2 METHODS

Findings presented are from a wider study of men's experiences of help seeking and everyday coping and management of mental distress (2015–2019). The overarching aim was to explore how a range of men of different ages and social backgrounds sought out help for troubles in living and the things that they did to positively manage and cope with emotional difficulties in their day‐to‐day lives. As part of exploring formal help seeking, men's use of support groups and what they perceived as effective and helpful in managing distress, was examined. Through a cross‐sectional, qualitative design, 19 men recruited from mental health support groups in South Wales, UK, took part in semi‐structured interviews. The study was given ethical approval from The School of Social of Sciences’ research ethics committee at Cardiff University.

Participants were recruited from a number of charity sector organisations and support groups that were aimed at people who may be experiencing distress such as depression and anxiety, also including loneliness and isolation. In accordance with the ethical approval clearance, NHS‐run groups were not recruited from. An internet search of local mental health support groups was conducted (both men's only and mixed support groups) and a sampling list was constructed. Facilitators who led groups were contacted by the researcher, explaining the research and asking if it would be possible to come along to talk to the group and distribute research flyers inviting men to participate in a research interview. Following this, male attendees self‐selected to take part in the research and contact details were exchanged to arrange a follow up interview.

The author, and sole researcher, carried out the semi‐structured interviews at either a private room within the support group organisation, or in a public location chosen by the participant. Interviews lasted between 45 min and 1.5 h. Written informed consent was gained in person prior to the interview. An interview schedule was used but merely as a topic guide rather than a script and the interviews were very flexible in nature. Participants were asked about their life and background, their experience of mental distress and why they came to attend the group, their experiences of attending the support group and in what way it helped them to manage their distress. Participants were also asked about their experiences of other forms of professional help seeking and what else they did (as well as attending the support group) to cope and manage emotional difficulties in their everyday lives. Participants’ sociodemographic information and health status was not officially collected using a questionnaire at the start of the interview, but the interview schedule ensured it asked about age, marital status, education, job occupation and experiences of physical and psychological health issues. All participants were offered a resources sheet of mental health services at the end of the interview. Participants were assigned pseudonyms at the interview stage to protect anonymity.

All interviews were audio recorded and transcribed. Transcripts were then imported into NVivo 10.2 and were analysed thematically (Braun & Clarke, 2006). An inductive and deductive approach to coding (Strauss & Corbin, 1990) was taken to allow the new, potential themes to emerge from the data without constraints but also with the aim to explore themes specific to men's experiences of utilising support groups for problems in living. Transcripts were read and reread by the researcher, with initial descriptive codes being applied and a coding frame being developed. The codes were then analysed and combined to create themes. The coding frame and any uncertainties with codes and constructed themes were reviewed with two supervisors to support the development of themes appropriately. Thematic maps were sketched to explore connections and relationships within or between themes and theme definitions were produced and validated by returning to check on the coded data and reread extracts. The aim was to interpret the meanings and significance underpinning each theme and move from a descriptive to an interpretative level. Coding and data analysis were carried out solely by the author with the coding frames, theme definitions, thematic maps, coding itself and data extraction being reviewed by the author's two research supervisors.

## 3 FINDINGS

Four core themes are presented and discussed below, reflecting participants’ experiences of using support groups for mental distress and what it is about support groups they perceived as particularly effective. As can be seen from Table 1, participants varied in age (29–74 years of age). All participants were white British. As it is widely assumed that men are reluctant to seek help for, and disclose experiences of distress, it may be surprising that findings from this study reveal that men are willing and able to discuss emotional difficulties, under the right circumstances, and they can, and do, benefit from local support groups. It is important to note, however, that this main finding of how men can benefit from support groups relates only to this small sample of men who have already attended a support group. Generally, men spoke about the positive aspects of the support groups they attended. There were two men from the same support group who said that one thing they disliked was the number of activities and guest speakers that were taking place at the group, instead of using the group for personal discussions with each other as a support group. As negative experiences were not frequently mentioned, the positive aspects of support groups for men's mental health will be the focus of these findings. The following themes illustrate the distinct aspects of support groups that can be beneficial for men experiencing distress and emotional difficulties.

**TABLE 1:** Description of support groups and participants

Description of support group	Participant pseudonym and age
Support group 1: Mixed gender, local community‐led support group for adults with mental health difficulties.	Thomas (62) Joseph (retired, 66) Mark (retired, 64) Jim (retired, 74) William (44) Adam (48)
Support group 2: A group for men to meet and do things together. To tackle isolation, loneliness and other difficulties. Men only.	Richard (24) Peter (retired, 62) Andrew (retired, 68) James (retired, 61) Michael (retired, 65)
Support group 3: Service that provides information, peer support groups, one‐to one counselling and training that promote the development of skills and strategies. Mixed services.	Samuel (29) Kyle (34)
Support group 4: A social informal peer‐support group for people experiencing mental health difficulties. Mixed service and activities.	Rhys (57)
Support group 5: User‐led open access support service that provides a drop‐in service. Mixed service.	Matthew (54) Ben (52) Albert (51)
Support group 6: National charity that provides a men's group for those experiencing mental health problems	Barry (38)
Support group 7: Football training session set up for people experiencing mental health problems.	Patrick (54)

### 3.1 ‘They've walked in my shoes’: The value of shared experience

One of the main factors of the support group setting that was commonly constructed as useful to participants was being able to be amongst others who had similar experiences and felt similar to them, which in turn led to a perceived level of understanding and empathy. This parallels with previous research that found that a support environment that offers a sense of shared understanding can be particularly appealing to men (Galdas et al., 2014).

Because you’re in a room with like‐minded people. Who’ve either been there or are there, and they can relate to what you say. (Mark, 64, Support group 1)

For Mark and other participants in the study, the ‘shared ownership’ created a sense of belonging in times of mental health distress. Belonging and companionship link social relationships to improved mental wellbeing (Thoits, 2011a), as described in Rhys’ account:

Rhys And I’d just feel good, speaking to people that sort of understood without me having to explain what was going on in my head. And that was the thing with [name of counsellor]. I felt like I needed to explain my symptoms that I’d been going through. Whereas when it’s peer support, it didn’t matter whether I did or not. I didn’t feel like I had to, but I go to meetings, if I don’t say anything that’s fine and nobody is going to say what’s wrong, you haven’t spoken.

Interviewer It’s like being with someone who resonates with how you’ve felt?

Rhys Yeah, they’ve walked in my shoes if you like. They might not have been to war but that doesn’t matter. It’s just like it doesn’t matter what’s happened because [name of the group] isn’t there to fix anything, we’re not medical, we’re not professional, we can’t fix anybody’s problems and nobody in [name of the group] fixed my problems, but they do just by being there. (Rhys, 53, Support group 4)

As noted in the above account, participants felt a connection with other people that they think are like them and this affiliation is a ‘deep, holistic understanding based on mutual experience where people are able to “be” with each other without the constraints of traditional (expert/patient) relationships’ (Mead, 2003:1). Experiencing distress can potentially be isolating and men spoke of the comfort and acceptance that was provided in knowing that there were others locally who had experienced similar to them:

Well, it’s made me feel less isolated because you know there are other people who are experiencing the same or very similar to you. Cause’ you can think you are the only one in the world experiencing these thoughts, doing these actions, but when you speak to the group you appreciate that other people have been there, maybe still are. […] I meant you’re talking to people, with empathy for your situation not just people with sympathy for your situation. You’re talking to people who’ve done that and wearing the t‐shirt, that’s what I mean by less isolation. I don’t mean physical isolation from people I mean understanding of different people. (Joseph, 66, Support group 1)

Having ‘been there’ themselves, similar others can recognise expressions of distress and support the person's emotions through empathetic understanding.

### 3.2 A safe space to offload in confidence

Participants valued the safe, trusting and confidential environment that support groups were available to provide. It was recognised that not all men have the options of disclosing personal distress to others, such as family or friends and so this alternative space was particularly helpful:

Its more just a chance for people to talk in confidence about, you know, what’s stressing them, what’s bothering them, stuff they feel they can’t talk about elsewhere and obviously the idea that it is in confidence, so it doesn’t go outside. (Kyle, 34, Support group 3)

Amongst family and friends, men may feel that they must maintain a certain set of masculine norms that limit emotional expression in certain contexts (Courtenay, 2000), for example, at work and being the provider at home. Confidentiality, and the absence of personal ties to others in the group, minimises any potential threat to masculine identity. Some men commented on how they would not disclose their emotions anywhere outside of support groups space, especially to a spouse or close friend:

I find the chat, not so much helpful for me now because I’m feeling better but when I was poorly it was great because you could offload rubbish, and the nicest thing about it was it was in confidence […] I don’t offload anywhere else. I don’t offload to my wife, my family, my friends and I certainly wouldn’t offload in a pub environment. If I used one. (Mark, 64, Support group 1)

The affirmation of confidentiality amongst similar others that support groups offer can facilitate men's participation in support groups.

### 3.3 A chance to receive and offer tailored support

Those experiencing illness, especially mental health difficulties, might find it difficult to obtain support best suited for them. People with shared experience and knowledge can offer specialised dimensions of support and being the one to provide that specialised support can also have significant benefits to psychological wellbeing, especially for men. Some men recognised the significance of playing an important role in the group that they attended.

And I’ve been coming ever since, and I got better, slowly got better. And anyway, every meeting you come in and they give you a chat and have you got something to say. I say, “well I’m fine”. I’ve been good for weeks but what I’m going to do is keep coming and if somebody want me to do a one‐to‐one with them up here ‐ sometimes, I used to bring some men up here on a one‐to one, to talk with me, about depression and I tried to explain to them, how I sort of beat it, what I do and listen to their problems. So, I just kept coming to help other people really. (Thomas, 62, Support group 1)

Reciprocity is an integral part of the process of peer support group settings, which differentiates it from ‘expert worker support’ (Repper & Carter, 2011:395). Furthermore, having the opportunity to support other men can act as on‐going therapy for men (Simpson & Richards, 2019), as identified in the quote above. In the extract below, Mark explicitly acknowledged how contributing to the group helped him, having gone back to the group despite no longer attending following recovery:

Mark I had a birthday card of the group and it said something like we “miss you at group, we need your guidance” or something like that. Which was very complimentary. So, I come now because I feel I can contribute rather than participate.

Interviewer And help them?

Mark Yeah, and it makes you feel better doesn’t it.

(Mark, 64, Support group 1)

Adopting a particular role within the group setting could be a way of reconstructing masculine identity in that particular community (Creighton & Oliffe, 2010) and allowed participants to preserve or reconstruct masculine status.

### 3.4 ‘I find coming here quite relaxing’: Reducing isolation and social benefits of groups

In addition to providing much needed mental health benefits, it was noted that support groups were helpful in providing support for other areas of life. Support group settings provided a space for social contact to build social and friendship networks as well as being a place to discover new interests. This seemed to be a deciding factor for Kyle in attending a support group:

You know, I didn’t have what you call a support network. When you go to the doctor, they always ask, you know, have you got a support network, that kind of thing. So, I suppose for me the obvious thing was I didn’t have a support network um, so [name of group] seemed like something to me that was worth trying. (Kyle, 34, Support group 3)

Some participants, due to personal circumstances, work or family estrangements, were in similar situations, and did not have a close support network to utilise in times of distress. In general, support group settings also served to meet new people, make new friends, keep busy and learn new things as well as gain other hobbies and interests.

Several of the men interviewed (nine out of nineteen interviewees) were either retired or unemployed, so attending a weekly support group provided structure to their week, something to look forward to and a chance to interact with others:

So, I find coming here is quite relaxing. It gets me out the house, it gets me socialising with people you know. I’ve met a lot more people here and with the legion than I would if I was sitting in the house all day. (Peter, 62, Support group 2)

It is the social element that the group provided that was particularly useful to Peter. For Andrew, the support group enabled him access to such social activities:

Basically speaking, it gets you out of the house and it gives you an interest, doesn’t it really. Which is good. It creates a kind of a balance really, you come here and all that and you take part in it. […] You’ve got to have an interest in something. Because there’s nothing worse than being bored. Boredom is devastating. (Andrew, 69, Support group 2)

The quotes above highlight the influence that social participation can have on mental health and health behaviours. Peter and Andrew may not have initially attended the group for specific mental health support but being surrounded by people in similar situations to them enabled them to informally seek help from a formal outlet and further facilitated them in managing any distress and future mental health difficulties they may face.

## 4 DISCUSSION

Support groups, or peer support, can provide an alternative to traditional forms of mental health assistance, such as medication or one‐to‐one therapy, as well as offering other social opportunities to men experiencing mental distress. The findings here identify how support groups present a safe place for people in similar situations to both offer and receive help. In parallel with the limited previous research that has examined men's support group use, the most effective aspect of a support group for the participants in this study was being amongst others with shared life experience of distress, which established mutual respect, understanding and empathy. Research has suggested that when it comes to improving depressive symptoms, peer support groups have similar efficacy to group cognitive behaviour therapy (CBT) (Pfeiffer et al., 2011). This is supported by the data here that discuss the men's preference for familiarity and companionship.

In presenting the findings, the paper explored the mechanisms through which social support and social groups can improve psychological wellbeing. Tailored support and relationships with others who have direct experiential knowledge seemed to enable group members to develop active coping and mutual understanding, which can be influential in shaping illness and healthcare experiences (Gage, 2013). Thoits (2011a) argues, through her concept of social mechanisms, that empathy from similar others is a factor that can reduce distress and emotional impacts. Previous research has found that in support group settings, the relationships established are based on a unique mutual understanding of experience, which cannot be developed with those who have not had that same experience (Gage, 2013; Thoits, 2011a, 2011b). The specific mental health‐related support that can be offered from others within a support group was also something participants perceived as effective. According to Mead et al. (2001), it is reciprocity that distinguishes peer‐to‐peer support group relationships from the unequal relationships and power dynamics that are found between a patient and professional. Being able to provide support to others facilitates a defining role within a group as provider, problem solver and someone in control, aligning with masculine ideals.

Participants also talked about developing a new role, identity and purpose within the support group setting and this combined with being able to provide specific knowledge and support can influence wellbeing and render benefits with respect to coping with distress. Knowing who we are to others provides purpose and meaning in life that then shields against anxiety (Thoits, 2011a: 148) and so specific role identities within the group can have positive effects on mental wellbeing.

For some men, establishing a new role within the support group setting can be a chance to reconstruct their sense of self and masculine identity (Addis & Mahalik, 2003; Mansfield, Addis & Mahalik, 2003). The support group can be defined as a community of practice (Creighton & Oliffe, 2010; Lave & Wenger, 1991) through which masculine identity is contextually reframed through stories of shared experience, shared identity and social positioning. Similar to Simpson and Richards’ (2009) study, this shared masculine identity that emerged in the support group settings allowed for the portrayal of a level of emotional vulnerability, something that may still feel unacceptable outside of that specific setting. Furthermore, it highlights the importance of social context in the construction of gender identities and the embodiment of masculinity more flexibly. In the support group context, being around other men with shared experience facilitates norms that enable masculinity to be reconstructed into more health positive ways.

For older men, adjusting to life following retirement may be challenging as many men focus on the importance of work throughout their adult lives and with retirement comes identity and role loss, reduced sense of purpose and disconnection from others (Kleiber & Linde, 2014; Oliffe et al., 2013). For some older and single participants, being part of a group and community provided participants with routine, ritual and contact which can act as a buffer against distress, isolation and disconnection while at the same time preserving masculine identity through a sense of belonging (Keohane & Richardson, 2018:8). Men's narratives surrounding their use of support groups and what they found useful shows that some men (of various ages) are able and willing to have emotional discussions with others (including other men), given the right context. Again, it is important to note that this relates to these men that are already attending a support group. A support group setting is artificially creating a social network and community of practice which works well in providing support, specific healthcare information, purpose, routine and companionship. All of which can contribute to lower levels of distress.

The findings have highlighted the value and significance of support groups for men who may be experiencing mental distress, from depression and anxiety to loneliness and social isolation. It is important to emphasise that this study does not claim that support groups are useful for all men, as what works for one man and his complex social circumstances may not necessarily be suitable for another. However, this paper highlights the potential for such environments to provide a safe space in which some men can disclose distress and be understood by others. GPs, nurses and other primary care professionals need to be aware of the local and various types of support groups that are available and utilise them as a nonclinical referral option. It may be that support services for mental wellbeing and distress, both for men and women and for men only, are lacking in all regions, locally and nationally. Or perhaps it is the advertisement and awareness of these support groups that is currently lacking. Understanding what men gain from support groups can be used to both market such groups in an effective way and to also empower group facilitators to accentuate these positive aspects of the groups they run. In targeting mental health support groups at men, services should emphasise that men are not alone in their experience and promote groups as opportunities to meet and connect to others who are experiencing similar distress within an informal, safe space.

There are some limitations to this study. The sample consists of men who are willing and able to speak openly about their distress, having had positive experiences of utilising support groups. It, therefore, misses out men who feel unable to disclose their distress to others. The findings, therefore, cannot be transferred to other men experiencing distress who may wish to attend a support group but have limited access to resources or those who may have attempted to attend support groups but encountered difficulty. In addition, the sample lacks diversity in social characteristics of sexuality and ethnicity, so overlooks the ways in which the experiences of men from these groups may differ and how such social identities might impact on engagement with mental health support. The findings discussed do not provide intervention research or outcome evaluation of successful peer support groups because it did not involve any measures of impact or any comparison with a similar group of people who have not attended support groups. However, the aims of the research were not to establish whether support groups do work but rather the positive ways in which they might work for men. What these findings have acknowledged, is the positive influence of support groups for some men, and the potential for such positive aspects to be accentuated and for more group‐based support to be developed as an alternative to other more costly and seemly inaccessible services, to benefit and support men experiencing emotional difficulties.

## CONFLICT OF INTERESTS

The author has no conflict of interest and confirm that this manuscript is not under consideration by any other journal.

## Data Availability

The data that support the findings of this study are available on request from the corresponding author. The data are not publicly available due to privacy or ethical restrictions.
